# Bone marrow stromal cell therapy improves survival after radiation injury but does not restore endogenous hematopoiesis

**DOI:** 10.1038/s41598-020-79278-y

**Published:** 2020-12-17

**Authors:** Miguel F. Diaz, Paulina D. Horton, Sandeep P. Dumbali, Akshita Kumar, Megan Livingston, Max A. Skibber, Amina Mohammadalipour, Brijesh S. Gill, Songlin Zhang, Charles S. Cox, Pamela L. Wenzel

**Affiliations:** 1 Children’s Regenerative Medicine Program, Department of Pediatric Surgery, McGovern Medical School, University of Texas Health Science Center At Houston, Houston, TX 77030 USA; 2 Center for Stem Cell and Regenerative Medicine, The Brown Foundation Institute of Molecular Medicine, University of Texas Health Science Center At Houston, Houston, TX 77030 USA; 3 Department of Integrative Biology and Pharmacology, McGovern Medical School, University of Texas Health Science Center At Houston, 6431 Fannin St, MSB 4.130, Houston, TX 77030 USA; 4 MD Anderson Cancer Center UTHealth Graduate School of Biomedical Sciences, Immunology Program, Houston, TX USA; 5 Department of Surgery, McGovern Medical School, University of Texas Health Science Center At Houston, Houston, TX 77030 USA; 6 Department of Pathology and Laboratory Medicine, McGovern Medical School, University of Texas Health Science Center At Houston, Houston, TX 77030 USA

**Keywords:** Haematopoiesis, Mesenchymal stem cells, Radiotherapy

## Abstract

The only available option to treat radiation-induced hematopoietic syndrome is allogeneic hematopoietic cell transplantation, a therapy unavailable to many patients undergoing treatment for malignancy, which would also be infeasible in a radiological disaster. Stromal cells serve as critical components of the hematopoietic stem cell niche and are thought to protect hematopoietic cells under stress. Prior studies that have transplanted mesenchymal stromal cells (MSCs) without co-administration of a hematopoietic graft have shown underwhelming rescue of endogenous hematopoiesis and have delivered the cells within 24 h of radiation exposure. Herein, we examine the efficacy of a human bone marrow-derived MSC therapy delivered at 3 h or 30 h in ameliorating radiation-induced hematopoietic syndrome and show that pancytopenia persists despite MSC therapy. Animals exposed to radiation had poorer survival and experienced loss of leukocytes, platelets, and red blood cells. Importantly, mice that received a therapeutic dose of MSCs were significantly less likely to die but experienced equivalent collapse of the hematopoietic system. The cause of the improved survival was unclear, as complete blood counts, splenic and marrow cellularity, numbers and function of hematopoietic stem and progenitor cells, and frequency of niche cells were not significantly improved by MSC therapy. Moreover, human MSCs were not detected in the bone marrow. MSC therapy reduced crypt dropout in the small intestine and promoted elevated expression of growth factors with established roles in gut development and regeneration, including PDGF-A, IGFBP-3, IGFBP-2, and IGF-1. We conclude that MSC therapy improves survival not through overt hematopoietic rescue but by positive impact on other radiosensitive tissues, such as the intestinal mucosa. Collectively, these data reveal that MSCs could be an effective countermeasure in cancer patients and victims of nuclear accidents but that MSCs alone do not significantly accelerate or contribute to recovery of the blood system.

## Introduction

Radiation injuries typically impact multiple organs, but hematopoietic failure is common even at low exposures^1^. At high doses, hematopoietic symptoms resulting from acute radiation syndrome (H-ARS) include severe neutropenia and thrombocytopenia leading to infection or bleeding, and ultimately death^2^. These are common clinical consequences of myeloablative conditioning that increase morbidity and negatively impact quality of life for patients undergoing treatment of hematological malignancies such as leukemias, lymphomas, and myelomas^3^. H-ARS is also a major global concern as the threat of radiological and nuclear disasters grows. Indeed, in preparation for such events, governments have supported stockpiling of medical countermeasures for treatment of victims of radiation incidents^4,5^. Hematopoietic stem cell (HSC) transplant is the most effective and appropriate therapy for H-ARS; however, suitable donors are unavailable for a large fraction of cancer patients and would be infeasible for victims in a mass causality event given the requirement for human leukocyte antigen (HLA) matching.

Cell-based therapeutics are available for numerous injuries and diseases, yet there is currently no FDA-approved cellular therapy for treatment of radiation injury^5^. Logistical challenges associated with delivery of a cell therapy in the aftermath of a mass casualty event necessitate ease of recovery from storage, safe and quick administration, and “off-the-shelf” compatibility^4^. MSCs have attracted attention as a potential universal cellular therapeutic, as immunological incompatibility is relatively innocuous to the recipient, MSCs signal through paracrine and systemic means thus broadly benefiting many organ systems, MSCs do not need to engraft long-term to exert reparative and immunomodulatory effects, and even non-viable MSCs have similar immunomodulatory capacity^6^. Further, clinical trials for other indications aimed at repair of multiple organs and tissues have demonstrated safety and benefit from MSC therapy^7^. MSCs have been found to secrete cytokines important for hematopoiesis and to promote engraftment of hematopoietic cells in experimental animal models, particularly when the dose of transplanted hematopoietic cells is low^8,9^. Ishikawa and colleagues showed that reactive oxygen species from damaged hematopoietic stem and progenitor cells are transferred to bone marrow stroma to prevent HSC senescence after 5-fluorouracil treatment^10^. Further, several reports have shown that transfer of mitochondria from bone marrow stromal cells to hematopoietic cells can impact survival and proliferation mechanisms. For example, mitochondrial transfer from bone marrow stroma to HSCs was shown during acute bacterial infection to facilitate rapid leukocyte expansion^11^. Mitochondrial transfer from bone marrow stroma has also been shown to protect acute myeloid leukemia cells and acute lymphocytic leukemia cells from chemotherapy by preventing apoptosis^12,13^. These properties make MSCs attractive candidates for alleviating multi-organ radiation syndromes typical of radiation accidents^4^ as well as treating any combined trauma-related injuries in connection with a disaster.

Available clinical and preclinical evidence supports that MSC infusion can help prevent hematopoietic graft failure when co-administered with hematopoietic cells^14^. More unclear is the effect of MSCs on endogenous hematopoiesis, their capacity for accelerating hematopoietic reconstitution, and their potential for restoring the blood system in the absence of a hematopoietic graft. Indeed, the majority of MSC research examining engraftment and hematopoietic recovery after myeloablation has utilized co-administration of hematopoietic stem and progenitor cells. Prior studies that tested the effectiveness of MSCs in promoting hematopoietic recovery after lethal irradiation over 30–60 day periods necessitated co-transplantation of hematopoietic stem and progenitor cells to prevent animal death, contributing to a deceptively positive outlook for hematopoietic recovery^15^. Other studies attempting hematopoietic readouts without co-infusion of hematopoietic grafts were limited to a 14 day period, which precedes the typical radiation-induced nadir in red blood cells and precedes the timeframe wherein hematopoietic recovery could take place^16^. We therefore sought to test the inherent efficacy of therapeutic MSCs in alleviating symptoms of H-ARS in an animal model of acute radiation injury by careful examination of the blood system and cells of the hematopoietic niche.

We found that MSC therapy improved survival after radiation injury, even in the absence of hematopoietic stem cell transplantation. The protective effect of the therapy does not appear, however, to originate from hematopoietic rescue or enhanced regeneration of the bone marrow. Radiation caused profound damage to the hematopoietic stem and progenitor cells in the bone marrow, resulting in pancytopenia. Hematologic parameters in the peripheral blood and frequency of hematopoietic stem and progenitor cells in the bone marrow were unchanged by MSC therapy. Radiation also depleted components of the hematopoietic niche in the bone, including mesenchymal stromal cells, pericytes, and endothelial cells. These cellular constituents of the niche were also unimpacted by MSC therapy. Gastrointestinal syndrome was evident from progressive weight loss, and histological examination of the gut revealed injury to crypts and intestinal epithelium. MSCs appeared to improve gut pathology and elevated growth factors with central roles in development and regeneration of the small intestine. Thus, we conclude that MSC therapy alone could be an effective countermeasure for use in cancer patients or radiological disasters, but that restoration of the blood system is not accelerated or improved.

## Results

### MSC therapy improves survival after radiation injury without altering hematopoietic composition in the periphery

**Figure 1:**
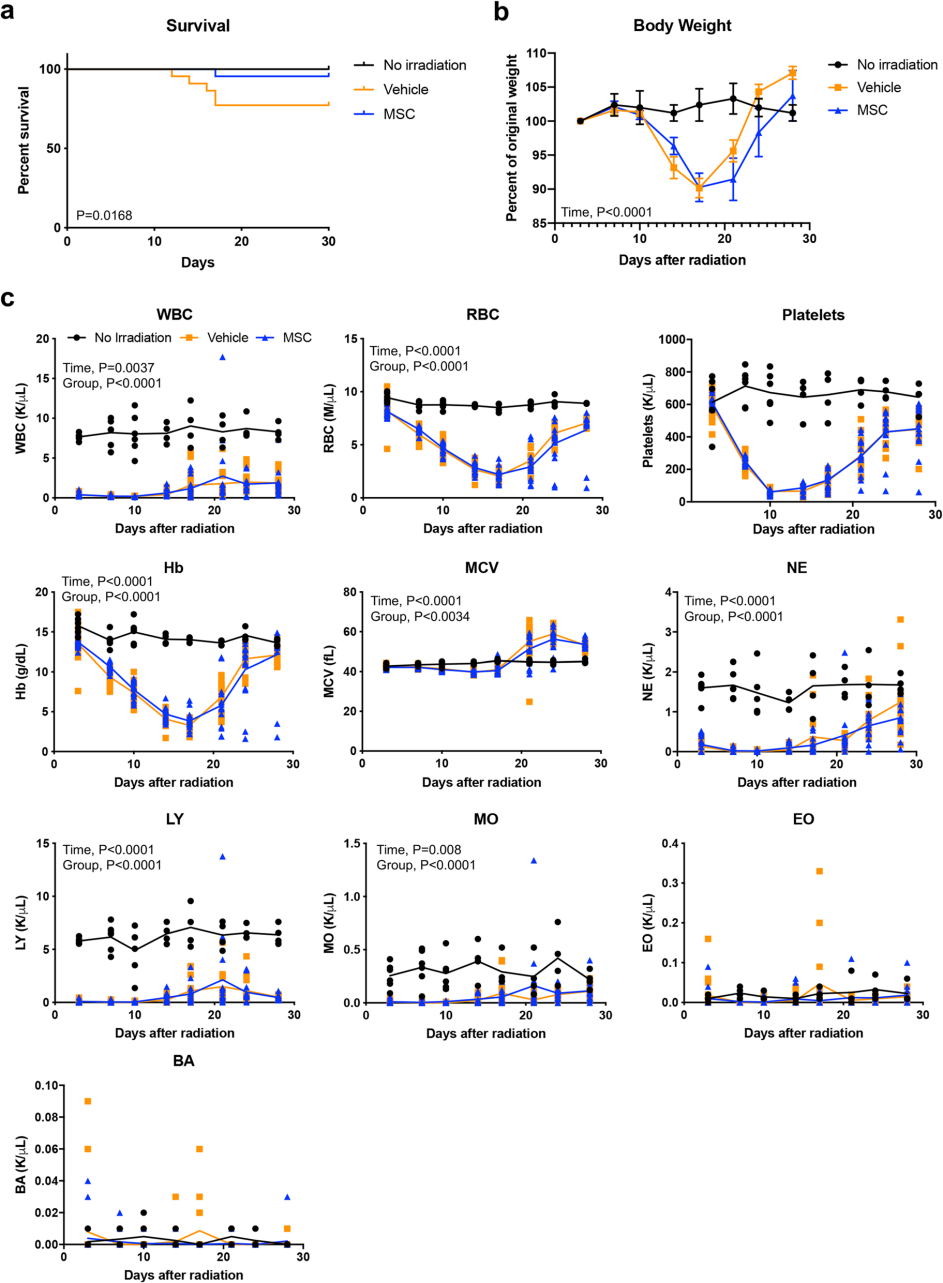
Radiation-induced pancytopenia persists despite improvement in survival of MSC recipients. (**a**) Survival following acute radiation injury is improved with MSC therapy 30 h after exposure (Kaplan–Meier survival plot; Log-rank test, *P* = 0.0168; n = 26 mice per group). Experiments were conducted across 6 independent experiments on different days. (**b**) Body weight drops in response to radiation, reaching lowest levels around 17 days (Two-way ANOVA, *P* = 0.2074 for treatment groups and *P* < 0.0001 for time; n = 20 mice per group). Experiments were conducted across 4 independent experiments on different days. Body weights are represented as mean ± SEM. (**c**) White blood cells were depleted within 3 days of radiation and remained low to the endpoint of the study. Platelets and red blood cells experienced a steady drop after 3 days, with nadirs at 17 and 10 days, respectively. Hemoglobin levels drop to a nadir of < 5 g/dL at 17 days after radiation exposure. Elevated mean corpuscular volume corresponds with red blood cell recovery beginning around 21 days after radiation but returns to near normal levels by day 30. Statistical significance of comparisons of treatment group and time by two-way ANOVA are shown on each graph. All data points are plotted for individual animals. *WBC* white blood cells, *RBC* red blood cells, *Hb* hemoglobin, *MCV* mean corpuscular volume, *NE* neutrophils, *LY* lymphocytes, *MO* monocytes, *EO* eosinophils, *BA* basophils.

We based our study design on a murine total body irradiation model developed by Orschell and colleagues, as it is the most well characterized model of H-ARS currently available and has been used over the past decade to screen over at least 50 medical countermeasures^17–19^. Adult mice received a single exposure to 8 Gy, a lethal dose for 30% of the cohort by day 30 (LD30/30). Mice began to die 12 days after radiation (Fig. 1a). Given the logistics of administering a cellular therapeutic in the context of a mass casualty event, we predicted that treatment would be unavailable within 24 h after radiation injury. Further, some reports have shown that the best time to rescue radiation injury is during the first 24–52 h^20^. We therefore administered 1.2 × 10^7^ MSCs/kg body weight, equivalent to approximately 300,000 MSCs per mouse, 30 h after irradiation using human bone marrow-derived MSCs validated by expression of seven surface markers (Supplementary Fig. S1). MSCs improved survival, with only 1 death among 26 animals (Fig. 1a). A significant drop in body weight coincided with the cluster of deaths between 12 and 17 days after irradiation (Fig. 1b). Lower body weights appeared to persist slightly longer when mice received MSC therapy and was likely due to prolonged survival of a few MSC recipients in poor condition.

A prior report also showed that murine MSCs could improve survival when administered alone within 24 h of radiation; however, the study did not determine whether hematologic parameters in the peripheral blood or bone marrow were altered^21^. The relatively low lethal dose in the present study enabled monitoring of hematopoietic recovery over a 30-day period. Thus, we examined complete blood counts every 3–4 days and found that leukocytes were rapidly lost within 3 days after irradiation. A nadir in platelets was detected between days 10–14 and in red blood cells at 17 days (Fig. 1c). Although individuals that received MSCs were significantly less likely to die, the hematopoietic system collapsed and hematological parameters were indistinguishable from those in the vehicle control mice (Fig. 1c). Two individuals that had received MSCs with persistent low erythrocytes and platelets beyond day 17 remained in poor condition but survived to the end point of the study at day 30. These data show that radiation-induced pancytopenia persists despite MSC therapy.

**Figure 2:**
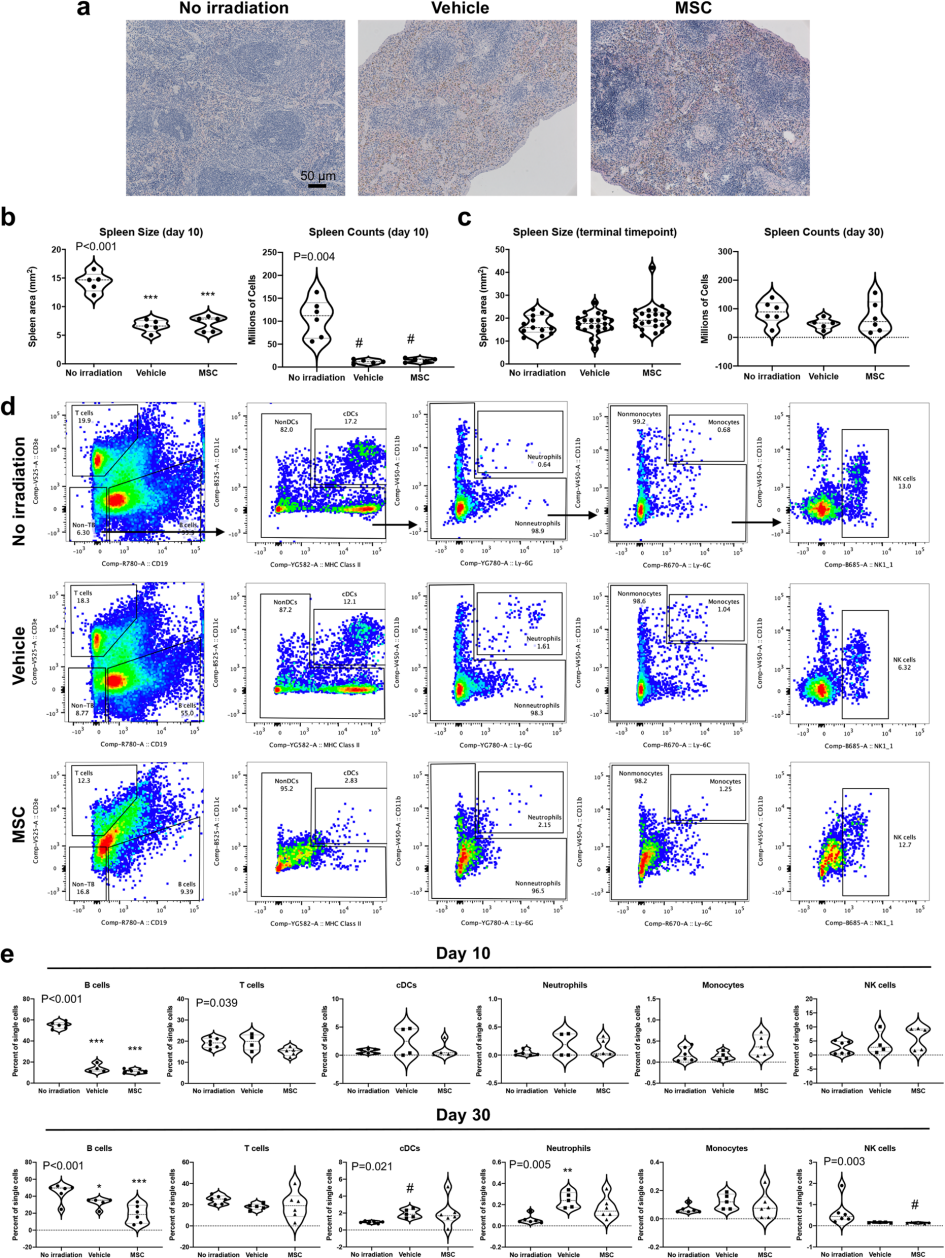
Radiation injures the spleen and alters immune cell composition. (**a**) H&E staining of the spleen at day 10 reveals fibrosis and visible hemosiderin, indicative of breakdown of red blood cells, in the irradiated mice regardless of whether they received vehicle or MSCs. (**b**) Splenic cellularity and size 10 days after irradiation are reduced but are indistinguishable in irradiated and unirradiated mice (**c**) at the terminal time point. (**d**) Representative flow cytometry plots of gating for immune cell lineages, including B and T lymphocytes, dendritic cells (cDCs), neutrophils, monocytes, and NK cells. (**e**) Quantification of splenic immune cell composition at day 10 and day 30. Statistical significance of differences between groups is depicted on the graphs. Posthoc comparisons are demarcated by asterisks *P < 0.05, **P < 0.01, and ***P < 0.001 for Holm–Sidak (One-way ANOVA) or pound symbol ^#^*P* < 0.5 for Dunn’s (Kruskal–Wallis One-way ANOVA) analyses relative to No irradiation control.

The radioprotective efficacy of some compound-based countermeasures have focused on splenic response to radiation^22^. Indeed, clinical data suggest that the spleen may modify hematologic toxicity of radiation, and splenic irradiation results in decreased spleen volume^23^. We therefore examined the spleen and found eosinophilic regions indicative of hematopoietic injury and decreased hematopoiesis at day 10 (Fig. 2a). We also found that spleen size was reduced approximately twofold at 10 days after radiation but recovered to normal size by the terminal timepoint at day 30 (Fig. 2b,c). Total spleen counts collected after red blood cell lysis revealed a far more profound loss of leukocytes (Fig. 2b); therefore, we analyzed immune cell composition of the spleen. B cells were severely depleted at day 10 and were not fully recovered by day 30 (Fig. 2d,e). The relative frequency of dendritic cells and neutrophils was significantly elevated in irradiated vehicle mice relative to unirradiated controls, although the higher variance in MSC recipients likely accounts for failure to detect a significant difference between this group and the unirradiated animals (Fig. 2e). MSC therapy did not appear to protect or significantly accelerate recovery of splenic hematopoiesis. In summary, these data suggest that MSC therapy after acute radiation injury can improve survival but that hematopoietic rescue in the periphery is an unlikely mechanism mediating the protective effect of MSCs.

### Bone marrow hypocellularity and architectural disruption persist despite MSC therapy

**Figure 3:**
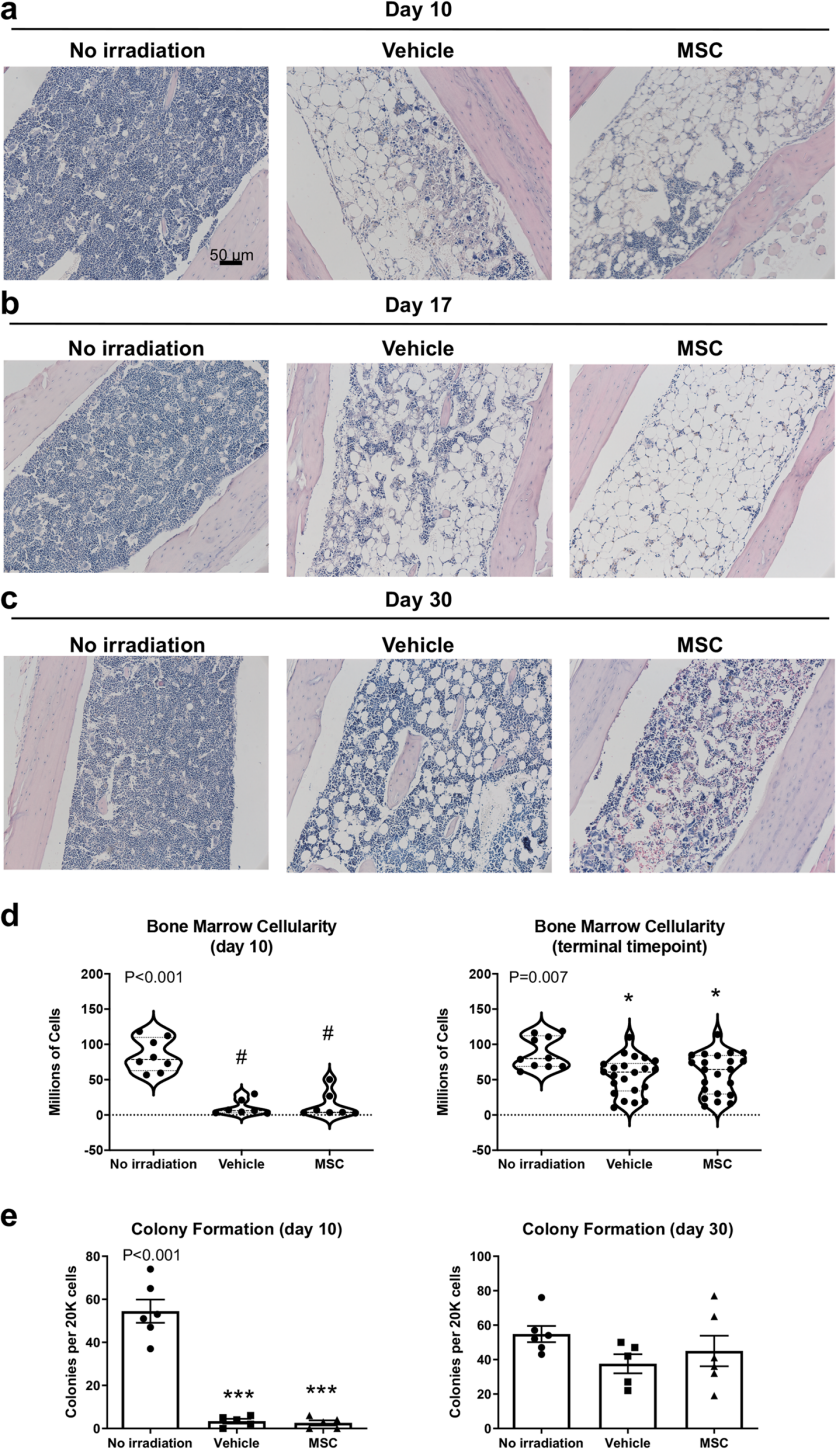
Bone marrow hypocellularity and architectural disruption is not abrogated by MSC therapy. (**a**) In the proximal tibia, extensive marrow aplasia and adipocytic expansion recognizable as empty round vacuoles are apparent 10 days after injury by H&E staining. (**b**) Mice that died between 14 and 17 days post-radiation exhibited severe hypocellularity of the bone marrow. Histopathology of two mice that died at day 17 suggests little evidence of hematologic recovery in the marrow. (**c**) Marrow is partially restored in surviving mice at day 30. (**d**) Recovery from aplasia was variable but largely reflected incomplete regeneration of the marrow at the study end point at 30 days. (**e**) CFU activity of whole bone marrow cells. Statistical significance of differences between groups is depicted on the graphs. Posthoc comparisons are demarcated by asterisks *P < 0.05 and ***P < 0.001 for Holm–Sidak (One-way ANOVA) or pound symbol ^#^*P* < 0.5 for Dunn’s (Kruskal–Wallis One-way ANOVA) analyses relative to No irradiation control.

The cause of improved survival was unclear from analysis of peripheral blood and spleen; thus, we examined the bone marrow in long bones of the mice. Histology of the proximal region of the tibia revealed widespread loss of hematopoietic cells and expansion of adipocytes in the irradiated animals as early as 10 days (Fig. 3a). Marrow appeared unchanged from the day 10 time point in two mice that died at day 17, one from the vehicle control group and the single mouse that died from the MSC therapy group (Fig. 3b). The single MSC recipient that died at day 17 was likely in poor condition due to a combination of hematological dysfunction and failure of other organs. Adipocyte expansion in the marrow tends to be enhanced where the hematopoietic compartment is depleted; thus, the observation by histology that adipocytes were numerous in the marrow of this animal supports that MSC therapy failed to promote hematopoietic recovery. Mice at termination of the study had residual signs of bone marrow aplasia and altered marrow composition in both the vehicle and therapy groups (Fig. 3c). Cellularity of femurs was reduced dramatically 10 days after irradiation (Fig. 3d). At 30 days, or at time of death, marrow cellularity had not fully recovered and was significantly lower than that of unirradiated mice (Fig. 3d). Analysis of hematopoietic function in colony formation assays showed profound reduction in capacity of whole bone marrow from irradiated mice to form colonies at day 10 (Fig. 3e). By day 30, hematopoietic activity was largely restored (Fig. 3e). These data suggested that MSC therapy alone is insufficient to restore bone marrow after irradiation, but we also sought to more precisely define the hematopoietic subsets that are important for homeostasis and stress response.

### Hematopoietic stem and progenitor cell deficiency is consistent with peripheral pancytopenia

**Figure 4:**
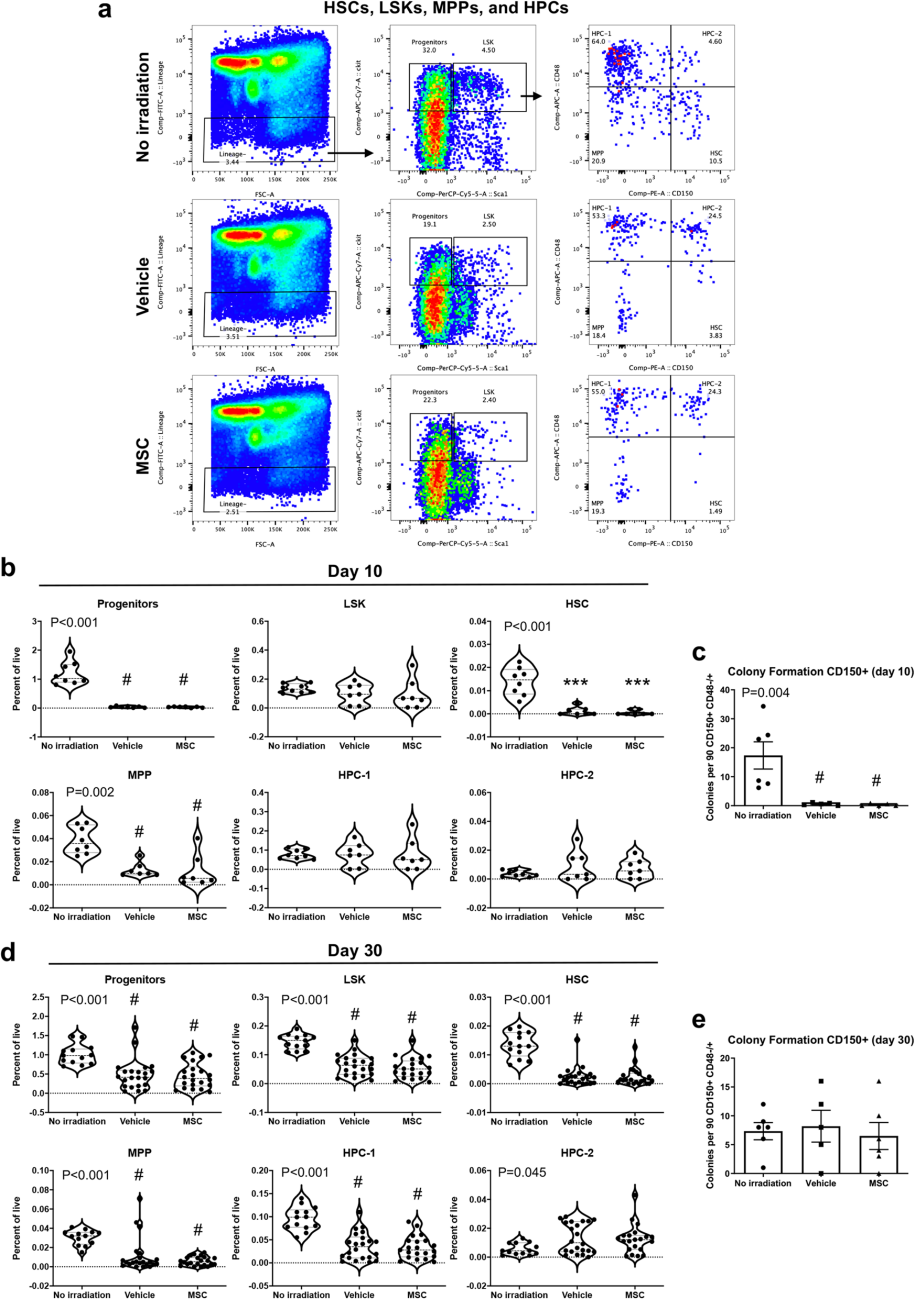
MSC therapy fails to promote recovery of hematopoietic stem and progenitor cells. (**a**) Frequencies of HSCs, LSK, HPC-1, HPC-2, MPP, and progenitors were determined by the depicted gating strategies. (**b**,**d**) Frequencies of hematopoietic subsets are equivalent in vehicle and MSC recipients. (**c**,**e**) CFU activity of sorted CD150^+^ cells. Statistical significance of differences between groups is depicted on the graphs. Posthoc comparisons are demarcated by asterisks ***P < 0.001 for Holm–Sidak (One-way ANOVA) or pound symbol ^#^*P* < 0.5 for Dunn’s (Kruskal–Wallis One-way ANOVA) analyses relative to No irradiation control.

Hematopoietic stem and progenitor cells are responsible for restoring the hematopoietic system after stress, so we asked whether the abundance of these populations was altered by MSC administration. Subpopulations with repopulating activity were measured using well established surface markers that can distinguish primitive long-term HSCs, multipotent progenitors, and various lineage-restricted progenitors (Fig. 4a; Supplementary Fig. S2; Supplementary Table S1). HSCs, MPPs, and progenitors were all reduced by irradiation in the vehicle and MSC therapy groups by day 10 (Fig. 4b). We also evaluated hematopoietic function of sorted lineage^-^ c-kit^+^ sca1^+^ CD150^+^ cells, which include long-term HSCs and HPC-2, as long-term HSCs (CD150^+^ CD48^−^) were rare in the irradiated groups. Importantly, this assay permitted examination of hematopoietic function on a cell-per-cell basis, since equivalent numbers were plated in colony formation assays. These assays show severe reduction in colony formation activity of CD150^+^ cells (Fig. 4c), suggesting that HSPCs are not only reduced in frequency but are also unable to effectively engage in hematopoiesis. Persistent reduction in these populations and in HPC-1 were apparent at the terminal time point in both groups (Fig. 4d), but hematopoietic function on a cell-per-cell basis was restored (Fig. 4e). Sca1 and c-kit expression levels have been reported to increase or decrease, respectively, in response to ionizing radiation^24^. We therefore evaluated HSC frequency using an alternate gating strategy independent of expression of the c-kit and Sca1 surface markers, which corroborated the reduction of HSCs at day 30 but was unable to discriminate between treatment groups at day 10 (Supplementary Fig. S3). Evaluation of the effects of sex on response to radiation injury and MSC therapy suggested modest to no differences between males and females in terms of hematopoietic cell frequencies and activity (Supplementary Fig. S4). Instead, these data indicate that males and females at the age analyzed are highly similar in their responses. Together, these data are consistent with previous studies that have shown decreases in frequencies of HSCs and hematopoietic progenitors with sub-lethal and lethal irradiation^25–27^. Given the central role that the HSC population plays in replenishing progenitors, mature blood cells, and specialized immune cells, these deficiencies are not surprising in light of the losses seen in the peripheral blood. This evidence is compatible with persistence of a functional hematological impairment despite MSC therapy, which was detectable within the peripheral blood as pancytopenia.

### Damage to the bone marrow niche is not abrogated by MSC therapy

**Figure 5:**
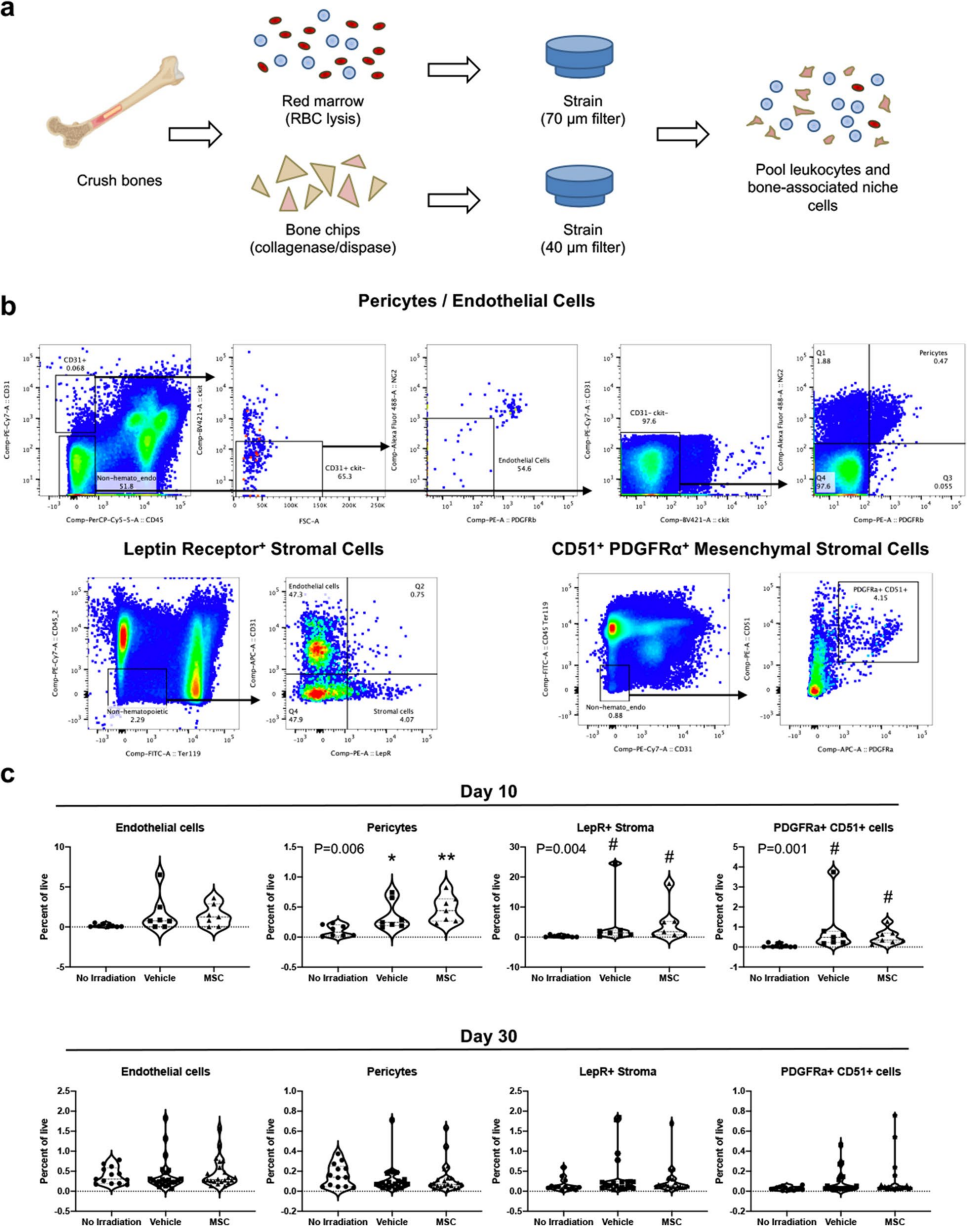
Cellular composition of the niche appears unchanged by MSC therapy. (**a**) Schematic overview of bone marrow isolation for analysis of hematopoietic and non-hematopoietic cells. (**b**) Representative flow cytometry plots show gating strategies used to define specified niche cell subsets. (**c**) Loss of hematopoietic cells from the marrow in irradiated mice results in greater relative frequencies of niche cells, although no significant differences exist between vehicle and MSC groups. Statistical significance of differences between groups is depicted on the graphs. Posthoc comparisons are demarcated by asterisks *P < 0.05 and **P < 0.01 for Holm–Sidak (One-way ANOVA) or pound symbol ^#^*P* < 0.5 for Dunn’s (Kruskal–Wallis One-way ANOVA) analyses relative to No irradiation control.

Radiation causes acute and long-term hematopoietic dysfunction in part by damaging the hematopoietic niche within the bone marrow^28^. Chief cellular elements of the niche include osteoblasts, stromal cells, endothelial cells, pericytes, adipocytes, and differentiated hematopoietic progeny. Perturbation of the frequency or function of niche cells can dysregulate retention and cycling of HSCs, leading to stem cell exhaustion and hematopoietic insufficiencies. We therefore examined the frequency of several niche cell populations and found expected relative increases in frequency due to depletion of hematopoietic lineages (Fig. 5a–c). MSC recipients exhibited no significant difference in the abundance of these populations relative to the vehicle control. There were no profound changes in the frequency of endothelial cells, pericytes, or LepR^+^ and CD51^+^ PDGFR-α^+^ stromal cell populations, although we cannot exclude functional alterations to the niche that exogenously administered MSCs could induce. Statistical analyses suggested modest differences between males and females in the frequency of niche cells, likely due in part to high variability in these populations across experimental groups. No clear biological trends in differential response were apparent, however, and none of the analyses at day 30 indicated evidence of sex as a relevant biological variable (Supplementary Fig. S4).

### Early administration of MSC therapy produces similar outcomes to 30 h infusion

**Figure 6:**
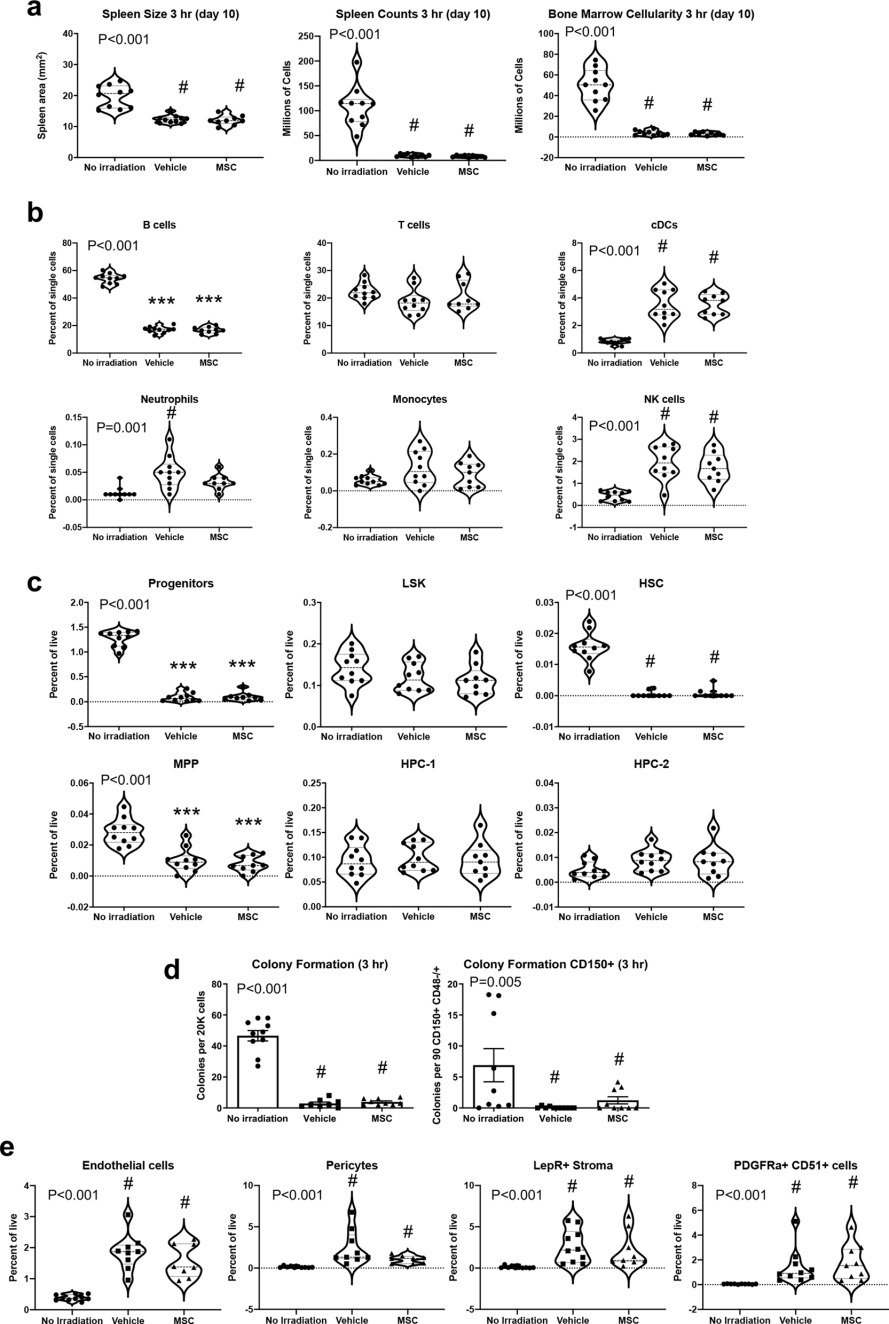
Administration of MSC therapy 3 h after radiation produces outcomes similar to 30 h infusion. Measurement of (**a**) spleen size and cellularity, (**b**) immune lineages in the spleen, (**c**) HSPC frequencies, (**d**) colony formation activity of whole bone marrow or sorted CD150^+^ cells, and (**e**) niche cell populations in the bone marrow. Statistical significance of differences between groups is depicted on the graphs. Posthoc comparisons are demarcated by asterisks ***P < 0.001 for Holm–Sidak (One-way ANOVA) or pound symbol ^#^*P* < 0.5 for Dunn’s (Kruskal–Wallis One-way ANOVA) analyses relative to No irradiation control.

A prior report suggesting that MSCs can rescue hematopoiesis employed therapy at 3 h after radiation exposure^29^. We therefore tested whether administration of human MSCs at 3 h could fundamentally alter the effects of radiation on hematopoietic injury and/or kinetics of recovery. All experimental conditions were identical, with the exception that cells were infused 3 h after irradiation. Spleen and bone marrow were collected at day 10, as hematopoietic defects were substantial at this time point in the 30 h therapy group. Size and/or cellularity of the spleen and bone marrow were not improved (Fig. 6a). Immune lineages in the spleen were equivalent in vehicle and MSC therapy groups, with the exception of neutrophil frequency, which was reduced in the MSC recipients (Fig. 6b). HSCs, MPPs, and progenitors were significantly reduced in vehicle and MSC marrow, as was observed in the 30 h recipients (Fig. 6c). Hematopoietic function was defective, as determined by colony formation assays with whole bone marrow and sorted CD150^+^ cells (Fig. 6d). Frequencies of hematopoietic niche cell populations were indistinguishable between vehicle and MSC groups (Fig. 6e). Consistent with our prior analyses of sex, mild statistical differences between males and females emerged for HPC-2, MPP, and progenitors, but no consistent differences could be identified to support the argument for improvement in any niche compartment specific to one of the sexes (Supplementary Fig. S4). In summary, MSCs fail to protect endogenous hematopoiesis regardless of whether administered immediately after radiation injury at 3 h or within a clinically feasible timeframe of 30 h. We conclude that MSC therapy alone could be an effective countermeasure for ARS but that benefit likely derives from non-hematological protection.

### Possible modifiers of improved survival with MSC therapy

**Figure 7:**
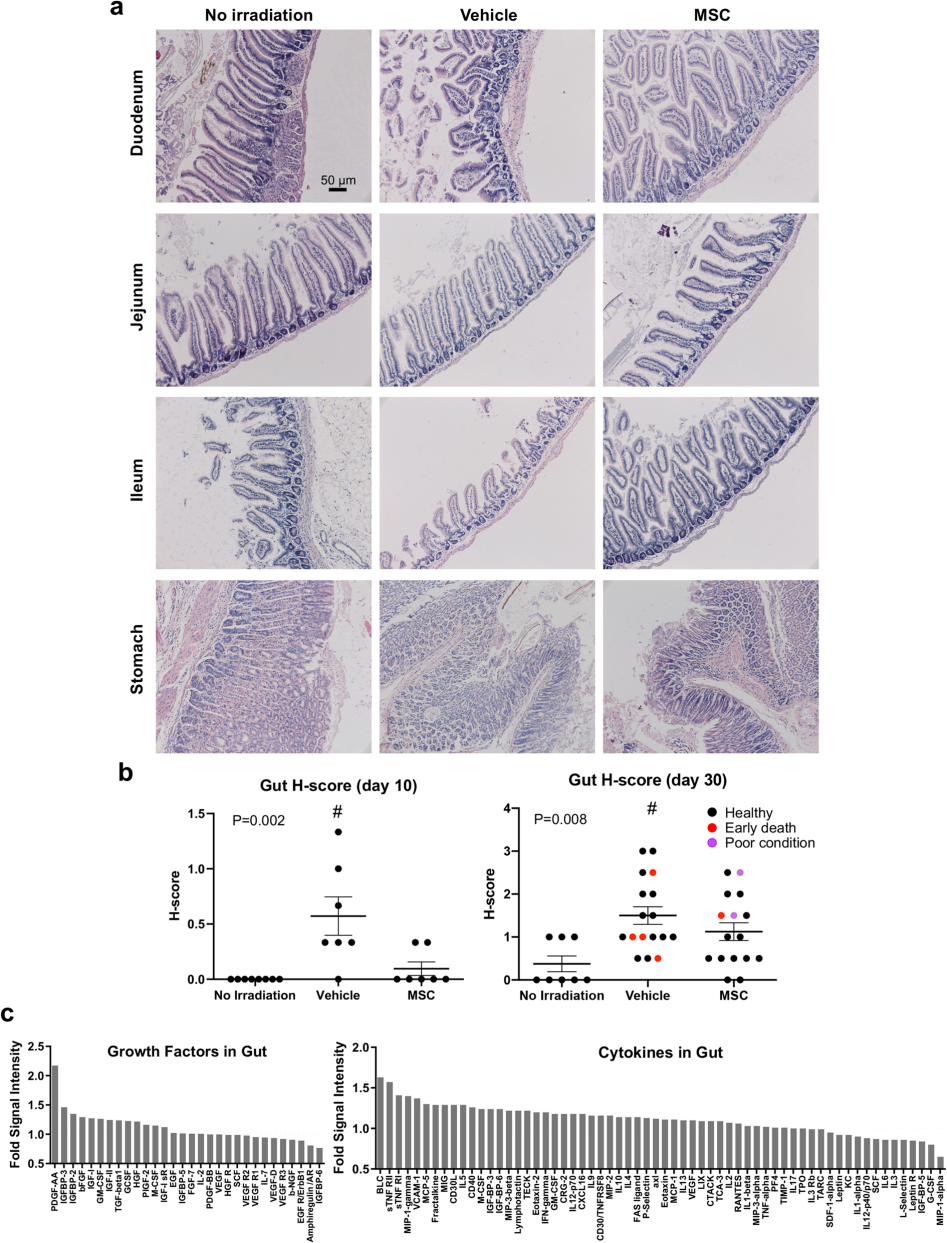
Small intestine exhibits signs of injury. (**a**) Photomicrographs of the small intestine show pathology of the mucosa. (**b**) H-scores of histopathological analysis of the gut at day 10 and the terminal time point are consistent with greater injury to the small intestine in vehicle treated mice that is less evident in recipients of MSC therapy (Kruskal–Wallis One Way Analysis of Variance on Ranks, Dunn’s Method ^#^*P* < 0.05 for No irradiation vs. Vehicle). (**c**) Cytokines and growth factors in the gut are altered by MSC therapy.

Radiation injuries typically involve high dose, heterogeneous exposure, and can negatively impact multiple organs, resulting in hematopoietic, gastrointestinal, and lung sub-syndromes of ARS^30^. Generally, gut injury is induced in radiation injury models at 8 to 12 Gy. Intestinal damage often corresponds with dramatic loss in body weight. The timing of maximal weight loss for irradiated groups in our study corresponded with deaths ranging from 12 to 17 days after radiation (Fig. 1a,b). Animals that lost approximately 25% of their original body weight died, with the exception of mice that received MSC therapy, some of which tolerated up to 33% weight loss. Assessment of the stomach, small intestine, and colon at day 10 and the terminal timepoint indicated that radiation had disrupted epithelial architecture and resulted in crypt dropout in the duodenum of the small intestine (Fig. 7a,b). Indeed, crypt loss was the principal contributor to the observed pathology. Consistent with a role for MSCs in modifying trophic signaling in damaged tissues, we observed a 2.2-fold increase in PDGF-A (Fig. 7c), a growth factor known to be essential for proper villus formation in gastrointestinal development. PDGF-A is secreted from intervillus epithelium to support organizing centers that stimulate proliferation and self-renewal of PDGFR-α^+^ mesenchymal cells in the center of the villus core^31^. Both PDGF-A and PDGFR-α-null mice die between embryonic day 10 and postnatal day 42^31^. Knockout mice manifest lethal gastrointestinal phenotypes that include lower abundance and abnormal morphology of intestinal villi, reduction of mesenchymal cells in the submucosa, and fewer specialized goblet cells which are necessary for production of the mucins that serve as a barrier against gut microbiota^31^. We suggest that elevated levels of PDGF-A in the small intestine of MSC recipients could be due to upregulated expression within the gut epithelium or could alternatively be due to greater numbers of surviving epithelial cells engaged in regenerating the mucosa. In the latter scenario, MSCs could promote recovery of the gut by protecting the epithelium and/or mesenchyme from cell death. Consistent with this possible mechanism, we also observed upregulation of two antagonists of TNF-α, soluble TNF receptor I and II (sTNF RI and sTNF RII), by 1.4- and 1.6-fold, respectively (Fig. 7c), which have both been shown to protect gastric epithelial cells from apoptosis induced by TNF in *Helicobacter pylori* infection^32^. Evidence in the literature also indicates that endothelial cell death drives mortality caused by radiation injury to the gut^33^. In fact, endothelial lesion develops in the lamina propria and within blood vessels of the adventitia before crypt stem cell damage, which is believed to trigger evolution of the gastrointestinal syndrome. Indeed, Lgr5^+^ intestinal stem cells that reside within intestinal crypts are believed to be dispensable for intestinal homeostasis but are absolutely essential for intestinal regeneration following radiation damage^34^. Some cytokines were elevated in the MSC therapy gut that play roles in Peyer’s patches. Specifically, B cell-attracting chemokine 1 (BLC) was increased 1.6-fold in the gut following MSC infusion (Fig. 7c). Because BLC is produced by the supportive cells in the Peyer’s patches that house B cell germinal centers, this could indicate that Peyer’s patch niches are preserved by MSC therapy. Lastly, increased insulin-like growth factor (IGF) signaling was apparent by increases in IGFBP-3, IGFBP-2, and IGF-1 by 1.5-, 1.4-, and 1.3-fold, respectively (Fig. 7c). Importantly, a recent report shows that IGF-1 is localized to pericryptal mesenchymal cells and that IGF-1 receptor is broadly expressed in crypt progenitor cells^35^. Following radiation injury of 12 Gy, IGF-1 signaling, presumably originating from the mesenchyme, stimulates mTORC1 to drive crypt regeneration via activation of facultative stem cells that replenish epithelium and replace lost crypt base columnar stem cells. Collectively, these data suggest that MSC therapy promotes signaling in the gut that reinforces intestinal regeneration after radiation injury.

**Figure 8:**
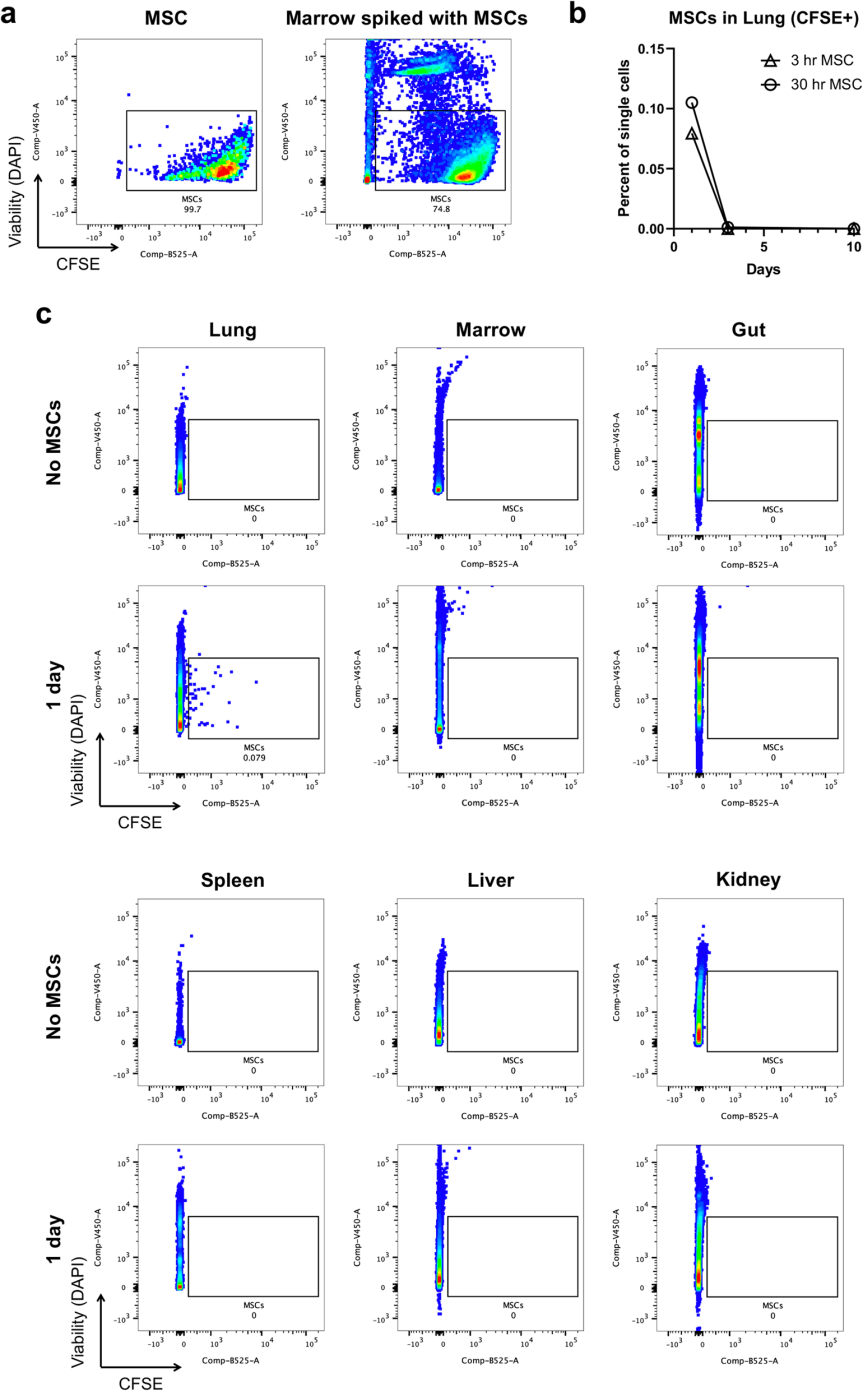
Human MSCs lodge in the lung and are undetectable in other organs. (**a**) CFSE-labeled human MSCs are detectable by flow cytometry when spiked into unlabeled bone marrow. (**b**) Lung was the only organ in which CFSE^+^ MSCs could be detected 1 and 3 days after infusion. This distribution occurred for administration of MSCs at 3 h and 30 h after irradiation. (**c**) Representative flow plots are depicted for all organs analyzed.

In a prior report, late development of fibrosis in the H-ARS mouse model was found to negatively impact the kidney, heart, and lung^36^. Yet, fibrosis generally manifested approximately 6–9 months after radiation exposure and continued to progress to 18–21 months. Our data cannot exclude alleviation of lung complications, renal pathology, or cardiovascular dysfunction associated with exposure to radiation, but the one-month timescale of our study makes these possible contributions to the improved survival seen with MSC therapy unlikely. Tracking of MSC distribution within various tissues of the body after infusion demonstrates that human MSCs are only present in the lung; thus, the effects MSCs have on other organs including the gut are likely via systemic signaling and/or global effects on the immune system (Fig. 8). Further study will be required to understand mechanistically how MSCs contribute to protection from the effects of radiation. Our data, however, do strongly suggest that the protective effect of MSCs on non-hematologic organs mediates the survival benefit of the therapy rather than any positive effect on restoration of the hematopoietic system.

## Discussion

Here, we provide direct evidence that MSC therapy reduces risk of death but does not ameliorate the effects of acute radiation on the blood system. Our work is distinct from prior studies in several ways: (1) the immunocompetent H-ARS model permitted evaluation of hematopoietic and immune cell lineages; (2) the intensity of radiation injury allowed monitoring of hematological parameters in the peripheral blood throughout the study period and at the traditional 30-day time point used for assessment of medical countermeasures; and (3) frequencies of niche cells were quantified rigorously by well-established surface markers. We find that pancytopenia in the peripheral blood persists despite MSC therapy and is characterized by collapse of all blood cells in the periphery to a level indistinguishable from untreated irradiated animals. In the bone marrow, hematopoietic stem and progenitor cells were no more abundant with administration of MSCs, nor were frequencies of various cell types thought to play critical roles in the hematopoietic niche altered, including LepR^+^ stromal cells, CD51^+^ PDGFR-α^+^ MSCs, pericytes, and endothelial cells. We conclude that MSC therapy improves survival not through overt hematopoietic rescue but by positive impact on other radiosensitive organ systems, including the gut. Collectively, these data indicate that MSCs could be an effective countermeasure for victims of radiologic accidents or cancer patients; however, MSCs alone do not significantly accelerate or contribute to recovery of the blood system.

The majority of research in the field looking at the effects of MSCs on engraftment and hematopoietic recovery have utilized coadministration of hematopoietic stem and progenitor cells, but a few have provided a glimpse of the potential of MSCs alone. Our study shows that administration of MSCs 30 h after acute radiation injury effectively minimized risk of death, with only 1 animal in 26 dying at day 17 of the 30-day period of the study. Comparison of this data to others is important as we consider the viability of this type of therapy as a medical countermeasure for a radiation disaster. For example, baboons cografted with CD34^+^ cells and MSCs have been shown to recover after radiation; whereas, baboons that receive MSCs alone do not recover^37^. Whether animals receive MSCs alone or MSCs with a hematopoietic graft, non-hematopoietic toxicity is considerable and suggests that application of adoptive cell therapy in this context is limited^37^. This is consistent with what is found in patients with hematologic malignancies undergoing autologous and allogeneic hematopoietic cell transplantation, for whom high-dose total body irradiation reduces relapse risk, but also causes increased, often fatal, gastrointestinal, hepatic, and pulmonary toxicities^38^. In contrast, in a study of NOD/SCID mice, administration of human MSCs expanded on soft substrates comprised of 1 kPa polydimethyl-siloxane (PDMS) was found to significantly improve animal survival relative to MSCs grown on more rigid surfaces such as tissue culture plastic or 100 kPa PDMS^39^. Relative to mice receiving no treatment, chance of survival after infusion of these mechanoprimed MSCs increased fourfold after an LD100/30 exposure. These findings are consistent with our own studies where we have found that preconditioning MSCs with force by application of shear stress impairs their ability to protect survival of mice after acute radiation injury, suggesting that external biophysical cues can profoundly alter the MSC secretome and function (unpublished data, PL Wenzel). An extension of the concept of a key role for soluble factors is reflected in another report that used an implantable device to separate lineage-negative donor bone marrow cells from direct interactions with the host marrow. Although this study was not focused on MSCs per se, they presented compelling evidence that soluble factor(s) were sufficient to rescue lethally irradiated mice by protecting endogenous HSCs^40^. Collectively, these data suggest that MSCs and/or the reparative factors they produce could be applied to enhance survival after ARS.

MSCs have been shown to improve hematopoietic engraftment and reduce graft failure, though little evidence supports acceleration of hematopoietic recovery. Mice in our study showed no improvement in complete blood cell counts and no indication that hematopoietic stem or progenitor cell frequencies in the bone marrow were altered by MSC infusion. Moreover, MSCs did not functionally improve hematopoietic activity when evaluated in whole bone marrow or on a cell-per-cell basis in sorted lineage^-^ c-kit^+^ sca1^+^ CD150^+^ cells in vitro. To date, no comparable studies looking at the efficacy of MSCs alone in treatment of radiation injury have carefully examined the composition of the bone marrow. In the study by Liu and colleagues showing that mechanoprimed MSCs can protect survival of NOD/SCID mice, authors conclude that complete blood cell count parameters are rescued but this is based upon use of a one-tailed t-test at only the terminal time point for white blood cells^39^. Similarly, modest improvements in blood counts were shown in a report that found MSC-derived extracellular vesicles when administered at 6, 24 and 72 h after exposure to 5 Gy non-lethal whole body irradiation could promote white blood cell and granulocyte recovery^41^. There were no other significant improvements in peripheral blood cell types, including monocyte and lymphocyte numbers. The most compelling data suggesting that MSCs can rescue endogenous hematopoiesis are from a study wherein umbilical cord blood (UCB)-derived MSCs were administered after ionizing irradiation^29^. Kim and colleagues found greater protective effects of UCB-MSCs relative to bone marrow-derived MSCs in in vitro assays of apoptosis; thus, they administered UCB-MSCs to mice shortly after exposure to X-ray irradiation. In this report of 6-week old C57BL/6 mice, exposure to 6.5 Gy led to 100% lethality; whereas, 43% of mice survived when infused twice at 3 h and 3 days with UCB-MSC. They also showed that UCB-MSCs trafficked to the bone marrow at remarkably high levels 3–6 days after tail vein injection (43% of the total cells recovered from marrow appeared to be CFSE^+^), improved peripheral blood counts, proliferation of hematopoietic cells in the marrow, and the frequency of the LSK population^29^. Differences between this report and the present study are that Kim and colleagues 1. administered cell therapy twice, whereas, we infused MSCs once, 2. utilized a level of X-ray irradiation that resulted in 100% lethality but our cesium-source ionizing radiation was lethal for only 30% of mice, and 3. irradiated 6-week old mice versus the 14–20 week old mice in the current study. This raises the possibility that infusion of multiple doses of cord-blood derived MSCs in younger mice exposed to severe myeloablation dramatically impacts hematopoietic outcomes. Beyond this study, the vast majority of preclinical studies showing improved animal survival with MSC therapy fall short of demonstrating consistent, compelling improvements in the kinetics of hematopoietic recovery and/or have incompletely characterized the hematopoietic compartment. The data from our study do not support a role for MSCs in accelerating recovery of endogenous hematopoiesis in the recipient, though our study design cannot exclude the possibility that other variables associated with radiation damage response and the cellular therapeutic itself play important roles in modifying hematopoietic regeneration. Such factors that remain unaddressed include the dose of irradiation, age of transplant recipients, strain of the mice, source of transplanted MSCs, number and timing of MSCs infused, and enrichment of MSCs for markers like c-kit or specific trophic properties. Further, long-term repopulation potential of HSCs exposed to radiation with or without MSC therapy has not been examined in our study. This functional assay of HSC transplantation into secondary recipient mice offers a more rigorous assessment of how MSCs impact HSC activity than the in vitro colony formation assays employed here. Likewise, the protective effects of MSCs on hematopoiesis could be revealed by repeated exposure to stress, such as a second radiation treatment or delivery of chemotherapeutic.

H-ARS is believed to be caused in part by loss of critical stromal and endothelial cell components of the hematopoietic niche, which hematopoietic cell transplantation is unable to restore^42–44^. Niche cells are believed to be lost, either by cell death, differentiation, or egress from the marrow, and their reduction in numbers has been detected over a decade after radiation injury^45^. This pathology is significant because the niche provides paracrine signaling and interactions with surrounding cell types that are necessary for hematopoietic regeneration and homeostasis. We present data that show administration of human MSCs does not alter the frequency of endothelial cells, pericytes, LepR^+^ stroma, or CD51^+^ PDGFR-α^+^ cells. Niche regeneration is required for hematopoietic recovery^42,43^ and, to date, no therapy currently exists to repair the hematopoietic niche of the bone marrow^46^. Given that defects in the bone marrow niche are associated with HSC exhaustion and cytopenias later in life that can cause morbidity and mortality, a future priority should be the identification of strategies capable of restoring these critical non-hematopoietic cellular components^17^.

The immunomodulatory, engraftment-promoting, and reparative properties of MSCs have been tested in an array of preclinical models and in clinical trials. The hematopoietic system is one of the most radiosensitive tissues in the body^47^ and when compromised can lead to devastating deficiencies in the body’s ability to deliver oxygen and fight infections. We show in a mouse model with a predictable dose response to irradiation, for which extensive assays and reagents are available to assess hematopoietic and MSC populations, that MSC-based cellular therapy can be administered as a countermeasure to reduce lethality after acute radiation exposure. Likely mediators of MSC efficacy are protection of non-hematological organs and moderation of inflammatory response. Thus, MSCs do improve survival but evidence does not support a role for MSCs in directly accelerating endogenous hematopoiesis of the host.

## Methods

### Radiation injury of mice

All experiments were approved by and conducted in compliance with guidelines from the Institutional Animal Care and Use Committee (IACUC) at the University of Texas Health Science Center at Houston. To ensure that our research design was rigorous and conducted according to established protocols, methods were modeled after a body of work by Orschell and colleagues^17–19^. The barrier facility housing the mice has a ^137^Cs source irradiator (GammaCell 40, MSD Nordion, Ottawa, Ontario, Canada). Irradiation and dosimetry for the current study was based upon the H-ARS model using the same irradiator available in our barrier facility^19^. Dose rates on this instrument were 0.88 Gy/min and were measured over the study period for all experiments. UTHealth Radiation Safety provided support for calculations of dose rates.

Equal numbers of male and female C57BL/6 mice were irradiated at 14–20 weeks old and body weights recorded. This permitted inclusion of both sexes equally across all treatment groups. Irradiation occurred at the same time each day as this variable has been found to impact sensitivity to radiation injury^19^. Mice were placed in a Plexiglas irradiation apparatus and exposed to a single uniform total body dose of gamma radiation of 8 Gy equivalent to LD30/30. At this dose, we expected survival in the control vehicle treated group to be 30% ± 20%^18^. Prior work established that 7.76–8.53 Gy total body irradiation in this model corresponds with LD50/30 and 7.96–8.72 Gy with LD70/30; however, these dose responses can be affected by a vast array of conditions, including handling and manipulation of mice during the acute phase^18^, so responses were confirmed independently with our instrumentation. Control mice were handled in a mock exposure by transfer into the Plexiglas irradiation apparatus and subsequently returned to cages. Groups of mice irradiated together were divided among treatment groups to ensure that each group received the same irradiation exposure conditions. Evidence in the literature shows improved survival with antibiotic support^19^; however, to eliminate inter-individual variability associated with different rates of consumption of antibiotics in food and water, mice received no antibiotics but received wet feed on days 4–30 post-exposure.

### Culture of human MSCs

Human MSCs used for cell therapy were derived from whole bone marrow from independent human donors (commercially obtained from AllCells, LLC, Alameda, CA). Cells were isolated and maintained as described previously^48^. Briefly, enriched mononuclear cells by phase separation in Ficoll-Paque were resuspended for immediate expansion in complete culture medium consisting of MEM-alpha (Thermo Scientific), 20% fetal bovine serum prescreened for quality in MSC differentiation and growth assays (Atlanta Biologicals), 100 units/ml penicillin (Gibco), 100 µg/ml streptomycin (Gibco), and 2 mM l-glutamine (Gibco). Nonadherent cells were removed after 2 days. Adherent colonies were expanded further and frozen at passage 1. Expression of cell surface markers defined by the International Society for Stem Cell Therapy, including CD90^+^, CD73^+^, CD45^-^, CD34^−^, HLADR^−^, CD19^−^, and CD11b^−^ were confirmed by flow cytometry^48,49^ (Supplementary Fig. S1). Thawed MSCs were plated at 1 × 10^5^ cells/ml, and medium was changed every 3 days.

### MSC therapy

Each group of mice irradiated together were divided randomly among treatment groups to ensure that each group received the same irradiation exposure conditions. Mice were treated with human bone marrow MSCs between passage 2–4 retro-orbitally at 3 h or 30 h post-irradiation, for a single administration of 1.2 × 10^7^ MSCs/kg body weight. Tracking experiments to determine tissue distribution in recipient mice included labeling of MSCs with 10 μM CFSE (carboxyfluorescein diacetate succinimidyl ester) for 15 min immediately prior to infusion, according to manufacturer’s instructions (BD Horizon). Mice were weighed every 3–4 days in conjunction with blood collection. Transplantation of human MSCs were approved by and conducted in compliance with guidelines from the IACUC and Biosafety Program at the University of Texas Health Science Center.

### Blood, spleen, and bone marrow processing

Peripheral blood isolation was performed as previously described^50^. Briefly, blood was collected in heparinized capillary tubes from the facial vein, then transferred to dipotassium EDTA-coated blood collector tubes for analysis of complete blood count (CBC) every 3–4 days on a Hemavet 950FS. At the terminal time point, blood was collected similarly, and white blood cells were enriched in the buffy coat by 1% dextran sulfate-PBS-EDTA separation followed by treatment with RBC lysing buffer (Sigma-Aldrich). Splenocytes were isolated for counting and phenotypic analysis by maceration through a 70-um strainer, lysis of erythrocytes with RBC lysing buffer for 7 min, centrifugation, and resuspension in 2% FBS-PBS. Long bones of both legs were recovered and cleaned of all muscle. Femurs and tibias used for flow cytometry were crushed in 2% FBS-PBS with a mortar and pestle (Fig. 5a). Cells in suspension from the red marrow were incubated with RBC lysing buffer and filtered through a 70-μm cell strainer. In parallel, bone chips were enzymatically processed for recovery of niche cells according to a prior report^51^, wherein chips were incubated for 45 min at 37 °C with agitation at 50 RPM in HBSS containing collagenase type II (1 mg/ml) and dispase I (0.2 U/ml). Cell solution from bone chip digestion was poured through a 40-µm strainer, centrifuged, and combined with red marrow previously treated with RBC lysing buffer above. Tibias were isolated from a subset of mice and were placed in 4% paraformaldehyde for pathological scoring. Fixed bones were processed through paraffin, sectioned, and stained by H&E for subsequent histopathological analyses.

### Dissociation of tissues for human MSC tracking

Tissues were dissociated to single cells for detection of CFSE-labeled human MSCs by flow cytometry. Lung was macerated by scalpel in chilled PBS, then incubated in 10 mM HEPES-PBS buffer containing Collagenase D (2 mg/ml) and DNase I (40 U/ml) at 37 °C for 45 min with agitation at 50 RPM, modified from a published report^52^. Cell suspension was filtered through a 70-µm strainer, centrifuged, and resuspended in 2% FBS-PBS containing DAPI. Liver and kidney were similarly minced by scalpel and incubated under the same conditions in HBSS containing collagenase type II (1 mg/ml) and dispase I (0.2 U/ml). Cells were strained through a 40-µm strainer, centrifuged, and resuspended in 2% FBS-PBS containing DAPI. Portions of gut tissue (2 cm each) were collected from three locations within the small intestine representing duodenum, jejunum, and ileum. Adipose tissue and digested food were removed and rinsed away in chilled PBS. Gut was cut into small pieces and suspended in calcium/magnesium-free HBSS containing EDTA (2 mM), collagenase VIII (1.5 mg/ml), and DNAse I (40U/ml), based upon modification of a published protocol^53^. Gut was incubated at 37 °C with agitation at 50 RPM for 45 min, then strained through a 100-µm strainer, centrifuged, and resuspended in 2% FBS-calcium/magnesium free HBSS with EDTA (2 mM) and DAPI.

### Histology and histopathological scoring

Spleens were collected and fixed in 4% paraformaldehyde at 4 °C overnight. Digestive tract tissue was recovered, including the stomach, duodenum, jejunum, ileum, and colon, and flushed with 50% ethanol/5% acetic acid in deionized water, according to a published protocol^54^. Length of the small intestine was removed, cut longitudinally, and rolled around a toothpick for “Swiss roll” sections. Tissues were fixed overnight in 4% paraformaldehyde-PBS at 4 °C and subsequently processed through paraffin, sectioned, and stained by H&E for subsequent histopathological analyses. Evidence of injury to the intestinal mucosa was determined in H&E stained sections of small intestine by a pathologist (S.Z.) blinded to the treatment group. The severity of radiation enteritis was determined by the degree of preservation of the epithelial architecture, crypt atrophy/dropout, fibrosis, metaplasia, villi branching, endothelial injury, and infiltration of inflammatory cells in the lamina propria. Together, these indicators of pathology were combined to result in the H-score, where 0 represents no obvious pathology and 4 is severe pathology.

### Flow cytometry

Subsets of hematopoietic and bone marrow niche cells were identified by immunostaining for flow cytometric analysis immediately following isolation of tissues. Antibodies for detection of HSCs, LSK, MPP, HPC-1, HPC-2, hematopoietic progenitors, endothelial cells, LEPR^+^ cells, PDGFR-α^+^ CD51^+^ stroma, pericytes, and immune cell subsets are outlined in Supplementary Table S1 and were selected based upon prior reports^55–59^. Unfixed cells were resuspended in 2% FBS-PBS buffer containing DAPI (1 µg/ml) or Ghost dye red 780 prior to analysis on a 3-laser BD LSR II flow cytometer, 5-laser BD Fortessa flow cytometer, or 5-laser BD Aria II sorter. Gating for all panels was determined with fluorescence minus one controls.

### Colony formation assays

Whole bone marrow was isolated as detailed above. Following RBC lysis and pooling of cells isolated from bone chips, viable cells were counted, and immediately placed into methylcellulose containing SCF, IL-3, IL-6, and EPO (Methocult M3434, STEMCELL Technologies) or processed for immunolabeling and sorting, then transferred to Methocult. Twenty thousand whole bone marrow cells or 90 lineage^-^ c-kit^+^ sca1^+^ CD150^+^ CD48^−/+^ cells were placed in 1.5 ml methylcellulose, sealed with humidity in a blood gas mix at 37 °C for 11 days, and subsequently scored for colony type and number.

### Cytokine and growth factor arrays

Small intestine was collected on day 3 from male mice exposed to 8 Gy that received vehicle control or human MSCs at 30 h after irradiation. Three 1.5 cm sections from the duodenum, jejunum, and ileum of each mouse were cut longitudinally, washed thoroughly in chilled PBS, and combined. Cell Lysis Buffer (RayBiotech, AA-LYS-10 ml) was prepared at 2× concentration with deionized water. Halt Protease and Phosphatase Inhibitor Cocktail EDTA free (100×; ThermoFisher) was added to yield working lysis buffer. Sections of intestinal tissue were added to chilled buffer, and lysis was facilitated by vortexing and use of a pellet mixer. At 30 min, the intestinal mucosa and submucosa was fully lysed and some muscle remained unlysed. Lysate was centrifuged to pellet tissue, then further centrifuged at 9500 rcf at 4 °C for 10 min to remove insoluble material. An aliquot was collected for protein quantification and lysate was stored at − 80 °C.

The RayBiotech Mouse Cytokine Antibody Array (AAM-CYT-3-2) and Mouse Growth Factor Array (AAM-GF-3-2) were analyzed simultaneously with paired samples. Manufacturer protocol was followed with some modifications for detection of IRDye 800CW Streptavidin (LI-COR) to enable improved specificity and lower background relative to HRP chemiluminescence. The recommended blocking buffer, Intercept (PBS) Protein-Free Blocking Buffer (LI-COR) was used with Tween-20 (0.05%). Array membranes were blocked for 45 min. Lysate was diluted in blocking buffer (diluted fivefold in blocking buffer as recommended before a final dilution in 1 ml). Each membrane was probed with 350 µg of lysate for 4.5 h at room temperature. After washing, array membranes were probed with the corresponding biotin-conjugated antibody (for cytokine or growth factor) overnight 4 °C on an orbital shaker at ~ 110 RPM. After washing steps, IRDye Streptavidin was prepared in blocking buffer at 1:2000 dilution in 2 ml volume. Array membranes were then probed for 45 min at room temperature. After washing, membranes were scanned on an Azure Sapphire Biomolecular Imager with 20 µm resolution and laser setting of 5. Analysis of the array images were performed using AzureSpot software and the RayBiotech Analysis Tool macro for Excel.

### Statistical analyses

Independent experiments were conducted on different days with primary human bone marrow cell lines. All data were analyzed with SigmaPlot 12.5 (Systat Software, San Jose, CA) for statistical significance and are reported as mean ± SEM. Parametric tests, including ANOVA with Holm–Sidak post-hoc comparisons, were used when data met assumptions of homoscedasticity and normality; otherwise, nonparametric tests such as Kruskal–Wallis one way ANOVA with Dunn’s Method were employed. Kaplan–Meier survival curves were evaluated by the log-rank test. Two-way ANOVA was used to compare changes in body weight, complete blood counts over time, and sex differences in mice across the three treatment groups.

## Supplementary information

Supplementary Information.

## Data Availability

The datasets generated and/or analyzed during the current study are available from the corresponding author upon request.
