# Publisher Correction: Bone marrow stromal cell therapy improves survival after radiation injury but does not restore endogenous hematopoiesis

**DOI:** 10.1038/s41598-021-95556-9

**Published:** 2021-08-04

**Authors:** Miguel F. Diaz, Paulina D. Horton, Sandeep P. Dumbali, Akshita Kumar, Megan Livingston, Max A. Skibber, Amina Mohammadalipour, Brijesh S. Gill, Songlin Zhang, Charles S. Cox, Pamela L. Wenzel

**Affiliations:** 1 Children’s Regenerative Medicine Program, Department of Pediatric Surgery, McGovern Medical School, University of Texas Health Science Center At Houston, Houston, TX 77030 USA; 2 Center for Stem Cell and Regenerative Medicine, The Brown Foundation Institute of Molecular Medicine, University of Texas Health Science Center At Houston, Houston, TX 77030 USA; 3 Department of Integrative Biology and Pharmacology, McGovern Medical School, University of Texas Health Science Center At Houston, 6431 Fannin St, MSB 4.130, Houston, TX 77030 USA; 4 MD Anderson Cancer Center UTHealth Graduate School of Biomedical Sciences, Immunology Program, Houston, TX USA; 5 Department of Surgery, McGovern Medical School, University of Texas Health Science Center At Houston, Houston, TX 77030 USA; 6 Department of Pathology and Laboratory Medicine, McGovern Medical School, University of Texas Health Science Center At Houston, Houston, TX 77030 USA

Correction to: *Scientific Reports,* 10.1038/s41598-020-79278-y, published online 17 December 2020

The original version of this Article contained an error in the author name Paulina D. Horton which was incorrectly given as Pau lina D. Horton. The original Article has been corrected.

