# Supplementary material for: Bone marrow stromal cell therapy improves survival after radiation injury but does not restore endogenous hematopoiesis
Source: Sci Rep. 2020 Dec 17;10:22211. doi: 10.1038/s41598-020-79278-y (PMC7747726; doi:10.1038/s41598-020-79278-y)
Supplement: Supplementary file 1 — Supplementary Information. [file 41598_2020_79278_MOESM1_ESM.pdf]

Bone marrow stromal cell therapy improves survival after radiation injury but does not restore endogenous hematopoiesis

Miguel F. Diaz[1,2,3], Paulina D. Horton[1,2,3,4], Sandeep P. Dumbali[3], Akshita Kumar[1], Megan Livingston[1,2,3], Max A. Skibber[1], Amina Mohammadalipour[3], Brijesh S. Gill[5], Songlin Zhang[6], Charles S. Cox, Jr.[1,2], and Pamela L. Wenzel[1,2,3,4,*]

[1]Children's Regenerative Medicine Program, Department of Pediatric Surgery, McGovern Medical School, University of Texas Health Science Center at Houston, TX, 77030, USA

[2]Center for Stem Cell and Regenerative Medicine, The Brown Foundation Institute of Molecular Medicine, University of Texas Health Science Center at Houston, TX, 77030, USA

[3]Department of Integrative Biology & Pharmacology, McGovern Medical School, University of Texas Health Science Center at Houston, TX, 77030, USA

[4]Immunology Program, MD Anderson Cancer Center UTHealth Graduate School of Biomedical Sciences, Houston, TX, United States

[5]Department of Surgery, McGovern Medical School, University of Texas Health Science Center at Houston, TX, 77030, USA

[6]Department of Pathology and Laboratory Medicine, McGovern Medical School, University of Texas Health Science Center at Houston, TX, 77030, USA

*To whom correspondence should be addressed

Supplementary Table S1. Antibody panels used for analysis of hematopoietic cell subsets and niche cell populations.

Mouse HSCs, LSKs, MPPs, and HPCs
CD150-PE (TC15-12F12.2, 1:100)
CD48-APC (HM48-1, 1:100)
Sca1-PerCP-Cy5.5 (E13-161.7, 1:100)
c-kit-APC-eFluor 780 (CD117, 2B8, 1:100)
Ter119-FITC (TER-119, 1:200)
B220-FITC (RA3-6B21:200)
Gr1-FITC (1A8, 1:200)
CD2-FITC (RM2-5, 1:200)
CD3-FITC (UCHT1, 1:400)
CD5-FITC (53–7.3, 1:400)
CD8-FITC (53–6.7, 1:400)
DAPI (1 µg/ml)

Leptin Receptor^{+} Stromal Cells
Leptin R-biotin (1:100)
CD31-APC (PECAM-1, 390, 1:200)
Ter-119-FITC (TER-119, 1:200)
CD45.2-PE-Cy7 (104, 1:200)
Streptavidin-PE (1:100)
DAPI (1 µg/ml)

Pericytes / Endothelial Cells
CD31-PECy7 (390, 1:400)
CD45-PerCpCy5.5 (30-F11, 1:400)
cKit-BV421 (2B8, 1:500)
NG2-Alexa Fluor 488 1:100)
PDGFRβ/CD140b-PE (APB5, 1:250)
PDGFRα-APC (APA5, 1:100)
Ghost dye red 780 (1:1000)

CD51^{+} PDGFRα^{+} Stromal Cells
mCD45.2-FITC (104, 1:200)
Ter119-FITC (TER-119, 1:200)
mCD31-PE-Cy7 (390, 1:200)
mCD51-biotin (RMV-7, 1:100)
mPDGFRα-APC (APA5, 1:100)
Streptavidin-PE (1:100)
DAPI (1 µg/ml)

Immune Cells in Spleen
Ly-6G-PECy7 (1A8-Ly6g, 1:100)
Ly-6C-APC (HK1.4, 1:100)
NK1.1-PerCPCy5.5 (PK136, 1:100)
MHC Class II-PE (I-A/I-E) (M5/114.15.2, 1:100)
CD11b-eFluor 450 (M1/70, 1:100)
CD11c-Alexa Fluor 488 (N418, 1:100)
CD19-APCCy7 (MB19-1, 1:50)
CD3e-BV510 (145-2C11, 1:50)

Supplementary Figure S1

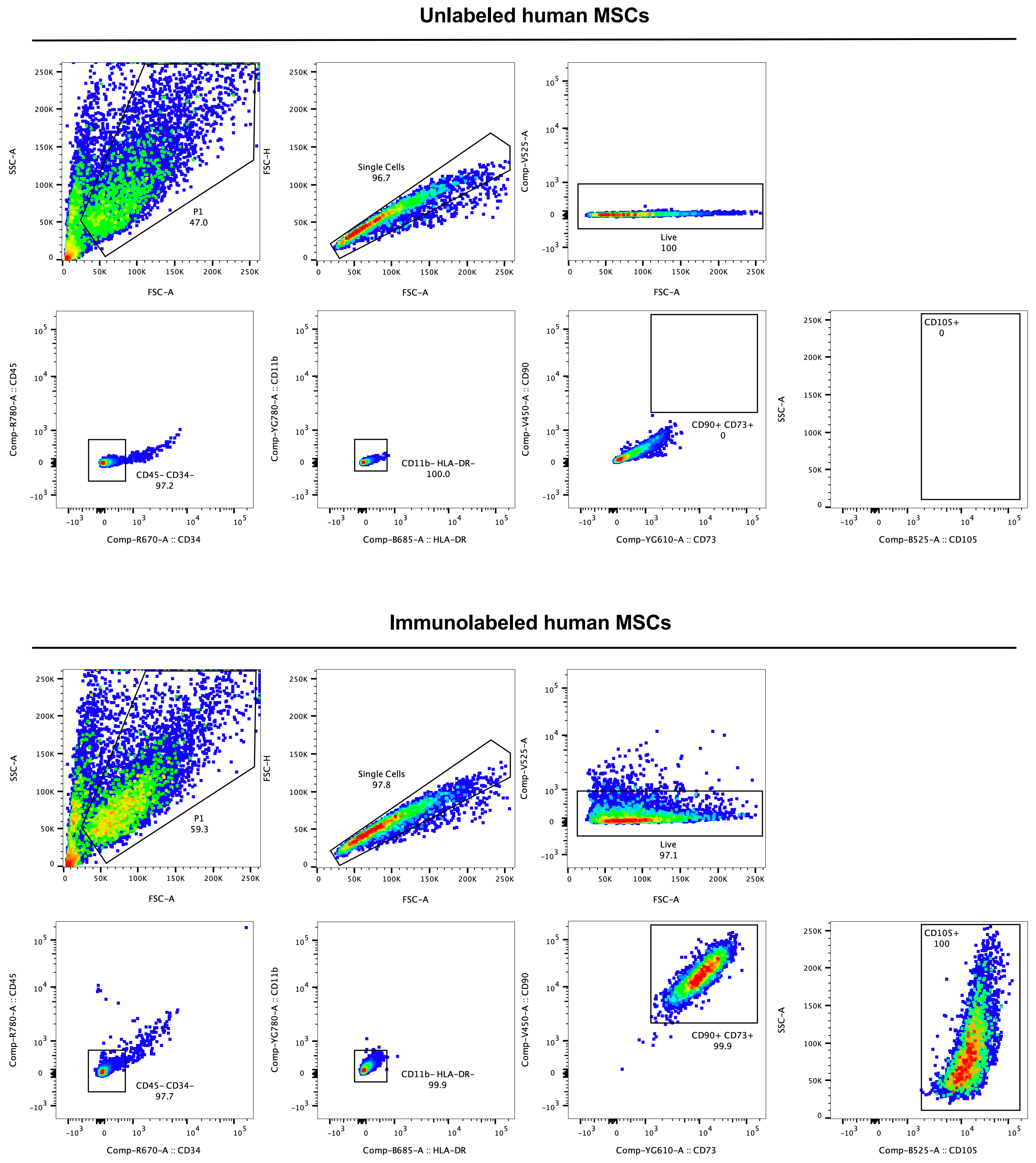

Supplementary Figure S1. Phenotypic markers validate identity of human MSCs.

Cells defined by surface markers as MSCs constitute 97.5% of cultured cells in the live gate. These cultures were used for MSC therapy of irradiated C57BL/6 mice.

Initial gating for all bone marrow panels

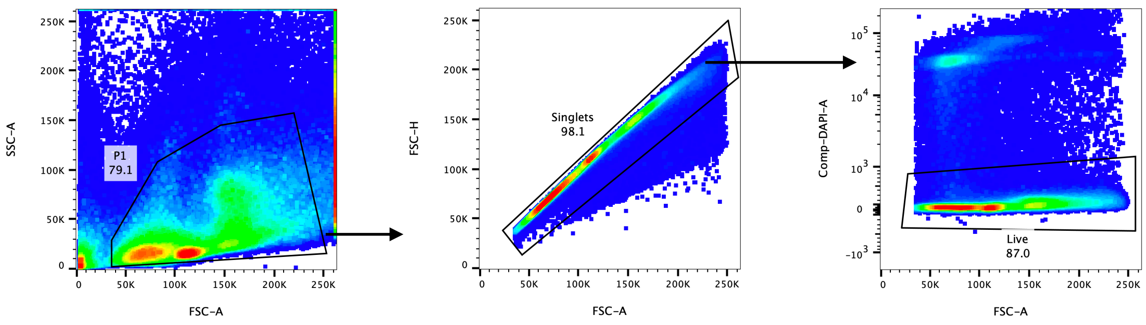

Supplementary Figure S2. Flow cytometry gating of marrow populations.

Plots depict initial gating used to select cells for all analyses of HSPCs and niche cells in the study.

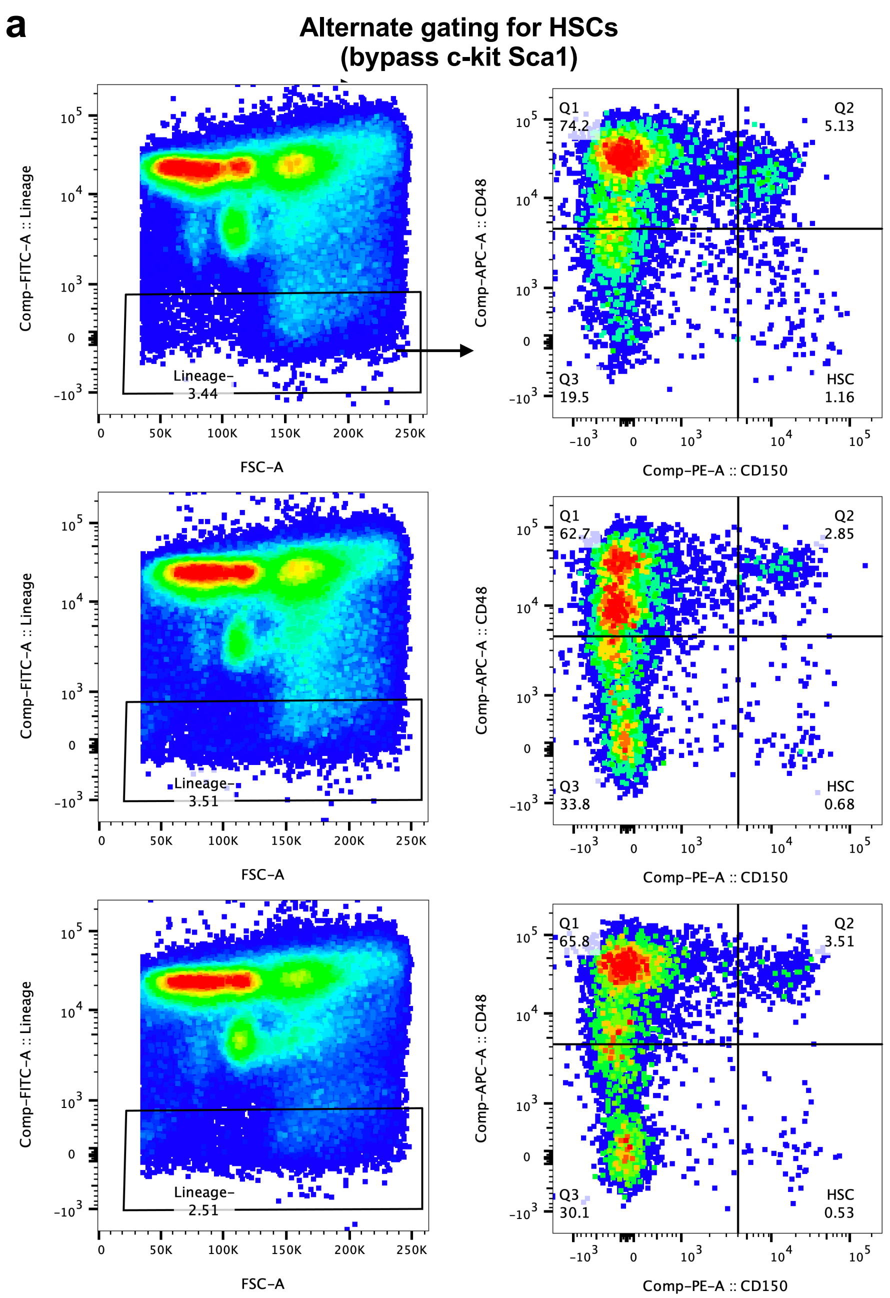
a
Alternate gating for HSCs (bypass c-kit Sca1)
Comp-FITC-A :: Lineage
FSC-A
Lineage-
3.44
3.51
2.51
Comp-APC-A :: CD48
Comp-PE-A :: CD150
Q1
74.2
62.7
65.8
Q2
5.13
2.85
3.51
Q3
19.5
33.8
30.1
HSC
1.16
0.68
0.53

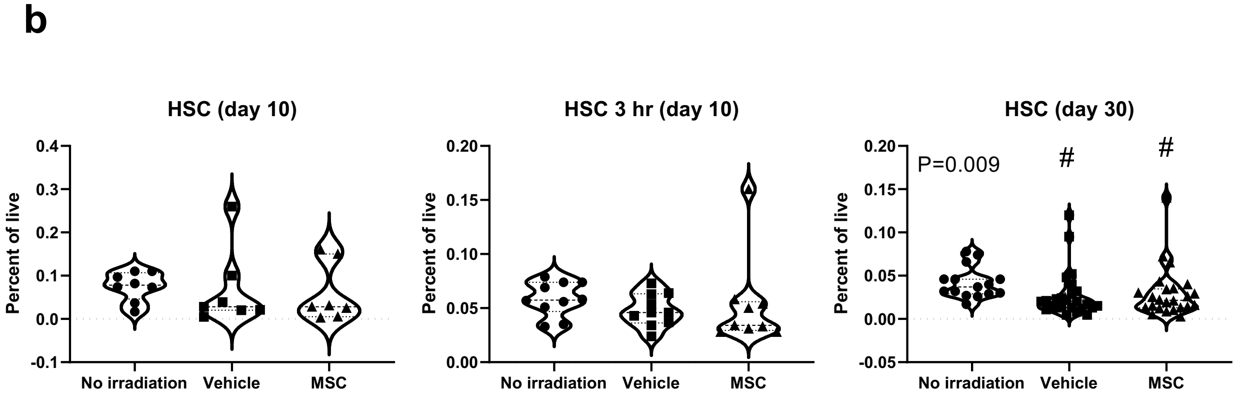
b
HSC (day 10)
HSC 3 hr (day 10)
HSC (day 30)
Percent of live
P=0.009
#
No irradiation
Vehicle
MSC

Supplementary Figure S3. Alternate gating of HSC population using only lineage markers, CD150, and CD48.

(a) Quantification of HSCs was conducted by an alternate gating strategy that was independent of c-kit and Sca1 expression and relied exclusively upon positivity for CD150 and negativity for CD48 and lineage markers. **(b)** HSC frequency at day 10 does not appear altered by radiation but is reduced at 30 days. This gating strategy does not appear to capture the reduction in HSC frequency that is detected by inclusion of c-kit and Sca1. Statistical significance of difference between groups is depicted on the graph. Posthoc comparisons are demarcated by a pound symbol # $p<0.5$ for Dunn's Method (Kruskal-Wallis One-way ANOVA) relative to No irradiation control.

Supplementary Figure S4

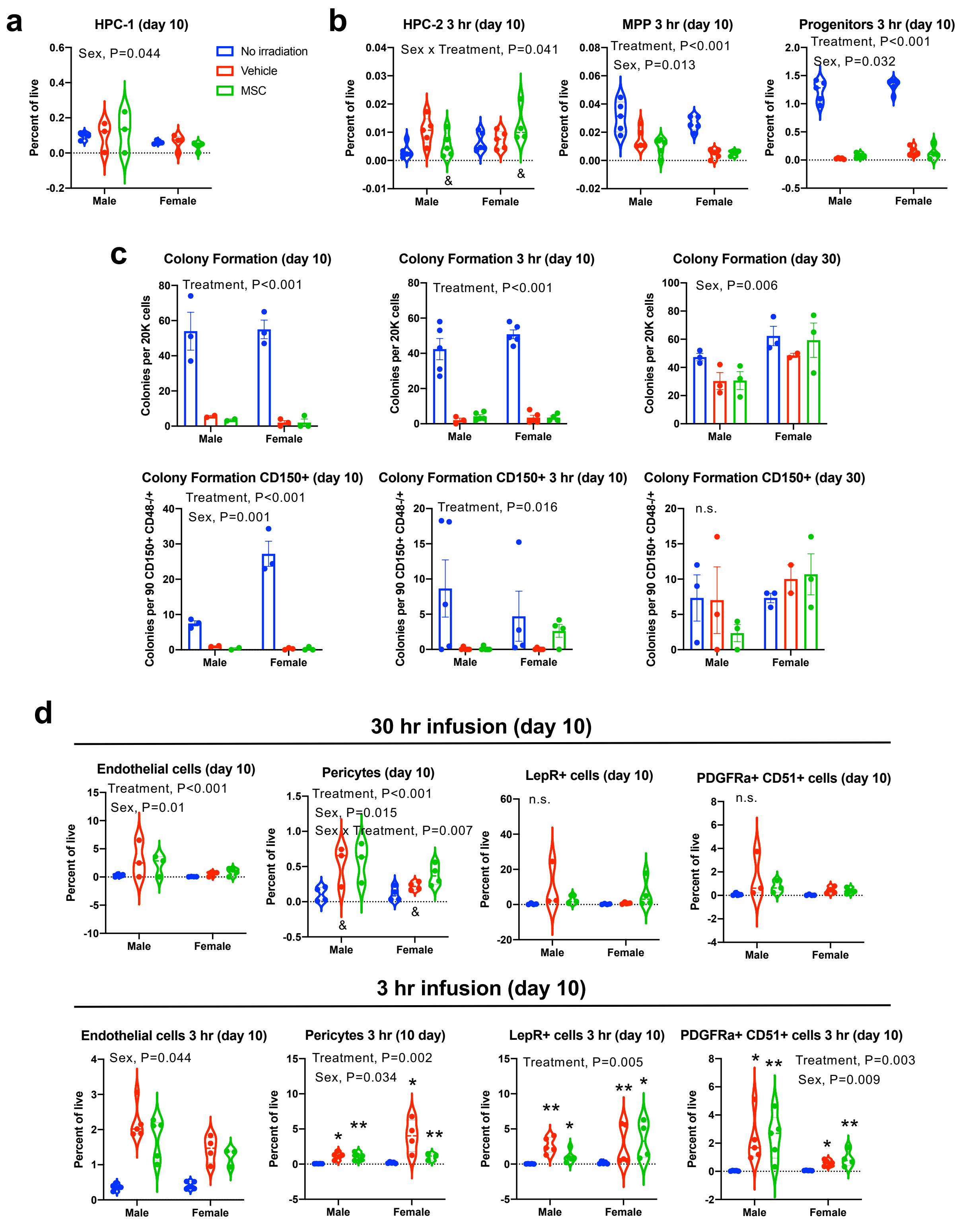

Supplementary Figure S4. Hematopoietic activity and frequencies of bone marrow cells in males and females do not point to reproducible effects of sex on response to irradiation or MSC therapy.

Statistical analyses of the effects of sex suggested some differences between males and females within experiments; however, no consistent patterns emerged between treatment groups across cohorts. **(a)** At day 10, only HPC-1 in the experiments designed to test efficacy of 30 hr MSC infusion showed some indication of a difference between males and females. Graphs of populations with no statistical indication of involvement of sex as a relevant variable were not included here. **(b)** HPC-2, MPP, and progenitors showed modest indication that males and females responded differently to radiation and therapy in the experiments measuring response to 3 hr MSC administration. **(c)** Colony formation assays with whole bone marrow (20K cells plated) and $CD150^+$ cells suggested variability in hematopoietic activity dependent upon sex, although the chief determinant was whether mice had received irradiation. **(d)** Statistical analyses also hinted at differences between males and females in the frequency of niche cells, likely due in part to high variability in these populations across experimental groups. None of the day 30 data showed evidence of sex as a relevant biological variable, as determined by Two-way ANOVA, and thus is not shown. Statistical significance of differences between groups is depicted on the graphs. Posthoc comparisons are demarcated by asterisks * $P<0.05$ and ** $P<0.01$ for Holm-Sidak analyses (Two-way ANOVA) relative to No irradiation control. The & symbol indicates a difference between males and females in (a) the MSC therapy group, suggesting a possible interaction between sex and therapy in HPC-2, and (d) the Vehicle control group, indicating possible interactions between sex and irradiation response in pericyte frequencies.
